# Exploring and applying genes to enhance the resistance to Fusarium head blight in wheat

**DOI:** 10.3389/fpls.2022.1026611

**Published:** 2022-10-27

**Authors:** Haigang Ma, Yongjiang Liu, Xueyan Zhao, Suhong Zhang, Hongxiang Ma

**Affiliations:** Jiangsu Co-Innovation Center for Modern Production Technology of Grain Crops/Jiangsu Key Laboratory of Crop Genomics and Molecular Breeding, Yangzhou University, Yangzhou, China

**Keywords:** wheat disease, *Fusarium graminearum*, Fusarium head blight, genetics, breeding

## Abstract

Fusarium head blight (FHB) is a destructive disease in wheat worldwide. *Fusarium graminearum* species complex (FGSC) is the main causal pathogen causing severe damage to wheat with reduction in both grain yield and quality. Additionally, mycotoxins produced by the FHB pathogens are hazardous to the health of human and livestock. Large numbers of genes conferring FHB resistance to date have been characterized from wheat and its relatives, and some of them have been widely used in breeding and significantly improved the resistance to FHB in wheat. However, the disease spreads rapidly and has been severe due to the climate and cropping system changes in the last decade. It is an urgent necessity to explore and apply more genes related to FHB resistant for wheat breeding. In this review, we summarized the genes with FHB resistance and mycotoxin detoxication identified from common wheat and its relatives by using forward- and reverse-genetic approaches, and introduced the effects of such genes and the genes with FHB resistant from other plant species, and host-induced gene silencing (HIGS) in enhancing the resistance to FHB in wheat. We also outlined the molecular rationale of the resistance and the application of the cloned genes for FHB control. Finally, we discussed the future challenges and opportunities in this field.

## Introduction

Fusarium head blight (FHB), which is also known as head scab and ear blight, caused by *Fusarium graminearum* (teleomorph *Gibberella zeae*) species complex is a fungal disease responsible for severe yield losses and poor grain quality in wheat (*Triticum aestivum* L.) (Bai and Shaner, 2004; Xu and Nicholson, 2009). The pathogen also produces mycotoxins such as trichothecenes and zearalenone contaminating infected wheat grains, which are harmful to humans and animals (Chen et al., 2019). Due to the warm temperature, abundant rainfall, and maize/wheat and rice/wheat rotations, the disease has been frequent and severe for the last decade worldwide, especially in China (Bai et al., 2018; Ma et al., 2019).

The most effective and economical solution for reducing FHB damage is to identify genes related to FHB resistance and apply them to breed disease-resistant varieties. The resistance to FHB is quantitative in wheat and no immune genes have been found so far (Bai et al., 2018). To date, a substantial number of quantitative trait loci (QTL) or genes conferring FHB resistance have been reported (Liu et al., 2009; Zheng et al., 2021). Previously, strategies and progress of wheat breeding for FHB resistance have been reviewed (Ma et al., 2019; Zhu et al., 2019). Here, we summarize advances in wheat resistance to FHB with the main focus on the characterized genes related to FHB resistance and their function in genetic improvement for the FHB resistance in wheat.

## Cloning resistance genes from common wheat using forward genetic approaches

### Cloning and utilization of *Fhb1*

Of the hundreds of QTL identified for FHB resistance by molecular mapping in common wheat, *Fhb1*, a QTL located on the short arm of chromosome 3B with the largest explanation of phenotype variation, provides durable and stable resistance to FHB. *Fhb1* candidate genes have been cloned recently using the map-based cloning approach. In 2016, a pore-forming toxin-like (*PFT*) gene was firstly cloned as the candidate of *Fhb1* (Rawat et al., 2016). However, this gene was also found in some susceptible accessions without *Fhb1* (Yang et al., 2005; He et al., 2018a; Jia et al., 2018). Before long, another gene named *HRC* or *His* was cloned as an *Fhb1* candidate by two independent studies (Li et al., 2019; Su et al., 2019). *HRC/His* encoded histidine-rich calcium-binding protein located in the nucleus. In comparison to that in the susceptible lines (*HRC/His-S*), the gene in the resistant lines carrying *Fhb1* (*HRC/His-R*) had a deletion in its genome, which is responsible for FHB resistance (Li et al., 2019; Su et al., 2019). The function of *TaHRC* was validated by using a BSMV-mediated gene editing system in Bobwhite and Everest (Chen et al., 2022a; Chen et al., 2022b).

It was recently found that *HRC/His-S* from *Leymus chinensis* (named *LcHRC* in the original article), which showed identical amino acid sequence to wheat HRC/His-S, bound calcium and zinc ion *in vitro* (Yang et al., 2020). *Arabidopsis thaliana* seedlings overexpressing *LcHRC* showed sensitivity to abscisic acid (ABA) (Yang et al., 2020). A protein that participates in heterochromatin silencing was identified as an LcHRC interactor through the screening of *Arabidopsis* yeast cDNA library (Yang et al., 2020). These results suggest a potential role of *LcHRC* in the regulation of genes involved in abiotic stress response. In wheat, HRC-S interacting proteins were identified through the screening of wheat yeast cDNA library (Chen et al., 2022b). One of the interactors, TaCAXIP4 [a cation exchanger (CAX)-interacting protein 4], was further validated to physically interact with HRC-S *in planta* (Chen et al., 2022b). The interaction with HRC-S suppressed TaCAXIP4-mediated calcium cation (Ca^2+^) transporting in yeast cells and resulted in reduced reactive oxygen species (ROS) triggered by chitin (Chen et al., 2022b), leading to the hypothesis that Ca^2+^ signaling-mediated ROS burst is essential for wheat FHB resistance. However, the details on how *HRC/His* affects FHB resistance remain equivocal, and more efforts are needed to elucidate their biological function and the regulatory network they mediated in defense response.

In fact, *Fhb1* locus has been widely used for FHB resistance breeding prior to the gene cloning. A large number of wheat varieties worldwide carried *Fhb1* locus, which confers moderate FHB resistance with the reduction of at most 50% in FHB severity (Bai et al., 2018; Zhu et al., 2019), which further confirmed the solid role of this locus in FHB resistance.

### Other resistance genes from common wheat

Besides *Fhb1*, many other QTL conferring FHB resistance of wheat have been reported, but the majority of their candidate genes remain unidentified. *QFhb.mgb-2A*, a major QTL located on chromosome 2A, was found in a recombinant inbred line (RIL) population, obtained by crossing an hexaploid line derived from a resistant cultivar Sumai3 and a susceptible durum cv. Saragolla (Giancaspro et al., 2016). Several genes, including *Fatty Acyl-CoA Reductase 1*, *Wall-associated receptor kinase 2* (*WAK2*), *Arginine decarboxylase*, *SWI/SNF-related matrix-associated actin-dependent regulator of chromatin subfamily A member 3*, and *Ubiquitin thioesterase otubain* genes, were detected in the QTL region (Gadaleta et al., 2019). The homeolog of *WAK2* in common wheat, which was named *TaWAK2A-800*, was identified later as a positive regulator of wheat resistance to FHB (Guo et al., 2021). Knocking down *TaWAK2A-800* in wheat using the virus-induced gene silencing (VIGS) method compromised FHB resistance, which may be attributed to the impaired defense pathway induced by chitin (Guo et al., 2021).

## Cloning genes with FHB resistance or mycotoxin detoxication from wheat using reverse genetic approaches

Decades of efforts in plant immunity have led to the development of plant resistance gene pool and the understanding of the mechanisms of plant disease resistance. The completion of wheat genome sequencing provides great convenience for the identification of the homologs of resistance genes in wheat through genome-wide homologous sequence analysis. Moreover, a variety of omics methods including transcriptomics, proteomics, and metabolomics will aid in identifying wheat resistance genes. A number of reverse genetics techniques are applied subsequently to overexpress and/or knock out/knock down the identified genes with the aim to verify their function. The widely used approaches in wheat include clustered regularly interspaced short palindromic repeats (CRISPR)/CRISPR-associated protein 9 (Cas9)-based genome editing, VIGS, and RNA interference (RNAi). They have been invaluable in analyzing gene function in wheat FHB resistance.

### Genes with FHB resistance in wheat

Many classes of genes have been implicated in the resistance to FHB in wheat. One group of them is referred to as pathogenesis-related (*PR*) genes. Increased expression of *PR* genes is a hallmark of plant defense response to pathogen attack. Based on gene sequence homology, two wheat *PR* genes, encoding chitinase (*PR3*) and β‐1,3‐glucanase (*PR2*), respectively, have been separately overexpressed in wheat and enhanced FHB resistance was observed in greenhouse but not in the field when using inoculated corn kernels (Anand et al., 2003). In another study, transgenic wheat lines with overexpression of wheat α-1-purothionin gene (a *PR* gene) exhibited increased FHB resistance in the field condition when using the spaying inoculation method (Mackintosh et al., 2007).

The essential role of plant hormones in the disease resistance is also a global consensus. Several genes involved in wheat phytohormone biosynthesis or signaling have been identified for FHB resistance. *EIN2* is a central regulator of ethylene (ET) signaling (Alonso et al., 1999). RNA interference (RNAi)-mediated *EIN2* silencing in wheat (Travella et al., 2006) reduced FHB symptoms (Chen et al., 2009), implying that ET signaling may promote wheat susceptibility to *F. graminearum*. Likewise, auxin was also implicated in FHB susceptibility of wheat. The expression of an auxin receptor gene *TaTIR1* was found to be downregulated during *F. graminearum* infection (Su et al., 2021). Knockdown of *TaTIR1* in wheat using RNAi technology increased FHB resistance (Su et al., 2021).

In addition to phytohormones, wheat metabolites are also essential for FHB resistance. Using a metabolomics approach, a research group identified several genes conferring resistance to FHB, including *TaACT* encoding agmatine coumaroyl transferase (Kage et al., 2017a), *TaLAC4* encoding laccase (Soni et al., 2020), and *TaWRKY70* and *TaNAC032* both encoding transcription factors (Kage et al., 2017b; Soni et al., 2021). These genes are all involved in the biosynthesis of hydroxycinnamic acid amides and phosphotidic acid, the major metabolites accumulated in wheat rachis after *F. graminearum* invasion. Suppressing the expression of these genes respectively using VIGS reduced FHB resistance.

Transcriptomics are powerful in identifying genes related to FHB resistance. Lots of genes whose expression are induced by *F. graminearum* have been identified by different methods, such as Genechips and RNA sequencing, and some of them have been validated to be effective in FHB control. A wheat orphan gene named *T. aestivum* Fusarium Resistance Orphan Gene (*TaFROG*), which is a taxonomically restricted gene specific to the grass subfamily *Pooideae*, was identified as an *F. graminearum*-responsive gene and promoted wheat resistance to FHB (Perochon et al., 2015). *TaFROG* encodes a protein with unknown function but binds to TaSnRK1α, which is a wheat α subunit of the Sucrose Non-Fermenting1 (SNF1)-Related Kinase1 and plays central roles in plant energy and stress signaling (Perochon et al., 2015). Another TaFROG interactor, *T. aestivum* NAC-like D1 (*TaNACL-D1*), which is a NAC [No apical meristem (NAM), *Arabidopsis* transcription activation factor (ATAF), Cup-shaped cotyledon (CUC)] transcription factor, was identified by using yeast two-hybrid screening (Perochon et al., 2019). *TaNACL-D1* was also responsive to *F. graminearum* and enhanced wheat resistance to FHB with unclarified mechanisms (Perochon et al., 2019).

Plant lectins, a class of proteins binding reversibly to mono- or oligosaccharides, are often associated with biotic and abiotic responses. Wheat genes encoding lectins have been shown to improve FHB resistance. *TaJRLL1* and *Ta-JA1/TaJRL53* are two genes in wheat encoding jacalin-related lectins. Suppressing their expression in wheat separately using VIGS compromised the disease resistance to FHB, while overexpressing *TaJRL53* in wheat enhanced FHB resistance (Ma et al., 2010a; Xiang et al., 2011; Chen et al., 2021).

Other genes, including *TaLRRK-6D* encoding a leucine-rich repeat receptor-like kinase (Thapa et al., 2018), *TaMPT* encoding a mitochondrial phosphate transporter responsible for transporting inorganic phosphate (Pi) into the mitochondrial matrix (Malla et al., 2021), *TaSAM* encoding an S-adenosyl methionine (SAM)-dependent methyltransferase that catalyzes the transfer of methyl groups from SAM to a large variety of acceptor substrates (Malla et al., 2021), *TaPIEP1* encoding transcription factor (Liu et al., 2011), and *TaSHMT3A-1* encoding serine hydroxymethyltransferase (Hu et al., 2022), are also identified to contribute to FHB resistance.

The abovementioned genes with FHB resistance are listed in **Table 1**. They varied enormously in gene products and biochemical functions. It seems that wheat utilizes extensive biological processes to defend against *F. graminearum* attack. As no immune genes were found in FHB resistance, a deep understanding of the signaling pathway mediated by these resistance genes will help to optimize wheat FHB resistance in breeding.

**Table 1:** Genes identified with FHB resistance in various common wheat variety.

Gene name	Wheat variety	Accession number	Gene products	References
*PFT*	Sumai3	included in KX907434.1^a^	pore-forming toxin-like protein	Rawat et al., 2016
*HRC*	Sumai3	MK450312^a^	histidine-rich calcium-binding-protein	Su et al., 2019
*His*	Wangshuibai	KX022629^a^	histidine-rich calcium-binding-protein	Li et al., 2019
*TaWAK2A-800*	Chinese Spring	TraesCS2A02G071800^b^	wall-associated kinase	Guo et al., 2021
*PR2*	Sumai3	undisclosed	β‐1,3‐glucanase	Anand et al., 2003
*PR3*	Sumai3	undisclosed	chitinase	Anand et al., 2003
*PR*	Undisclosed	X70665.1^a^	α-1-purothionin	Mackintosh et al., 2007
*TaEIN2*	Mercia	AL816731^a^	endoplasmic reticulum membrane-localized Nramp homolog	Travella et al., 2006 Chen et al., 2009
*TaTIR1*	Sumai 3	TraesCS1A02G091300^b^	putative auxin receptor	Su et al., 2021
*TaACT*	Wuhan-1	KT962210^a^	agmatine coumaroyl transferase	Kage et al., 2017a
*TaLAC4*	Sumai 3	MT587562^a^	laccase	Soni et al., 2020
*TaFROG*	CM82036	KR611570^a^	orphan protein with unknown function	Perochon et al., 2015
*TaSnRK1α*	CM82036, Xiaoyan 22	KR611568^a^, TraesCS1A02G350500^b^	SNF1-related kinase 1	Perochon et al., 2015 Jiang et al., 2020
*TaJRLL1*	CM82036	HQ317136^a^	jacalin-related lectin	Xiang et al., 2011
*Ta-JA1/TaJRL53*	H4564, Chinese Spring	AY372111^a^	jacalin-related lectin	Ma et al., 2010a Chen et al., 2021
*TaLRRK-6D*	Chinese Spring	TraesCS6D02G206100^b^	receptor-like kinase	Thapa et al., 2018
*TaMPT*	Chinese Spring	TraesCS5A02G236700^b^	mitochondrial phosphate transporter	Malla et al., 2021
*TaSAM*	Chinese Spring	TraesCS2A02G048600^b^	methyltransferase	Malla et al., 2021
*TaSHMT3A-1*	Chinese Spring	TraesCS3A02G385600^b^	serine hydroxymethyltransferase	Hu et al., 2022
*TaWRKY70*	Wuhan-1	KU562861^a^	WRKY transcription factor	Kage et al., 2017b
*TaWRKY45*	Chinese spring	AB603888^a^	WRKY transcription factor	Bahrini et al., 2011
*TaNAC032*	Sumai 3	MT512636^a^	NAC transcription factor	Soni et al., 2021
*TaPIEP1*	Shannong0431	EF583940^a^	ERF transcription factor	Dong et al., 2010; Liu et al., 2011
*TaNACL-D1*	CM82036	MG701911^a^	NAC transcription factor	Perochon et al., 2019
*TaUGT3*	Wangshuibai	FJ236328^a^	uridine diphosphate-glycosyltransferase	Ma et al., 2010b; Pei, 2011 Chen, 2013
*TaUGT5*	Sumai 3	TraesCS2B02G184000^b^	uridine diphosphate-glycosyltransferase	Zhao et al., 2018
*TaUGT6*	Sumai 3	TraesCS5B02G436300^b^	uridine diphosphate-glycosyltransferase	He et al., 2020
*Traes_2BS_* *14CA35D5D*	Apogee	MK166044^a^	uridine diphosphate-glycosyltransferase	Gatti et al., 2018
*TaABCC3*	CM82036	KM458975^a^, KM458976^a^	ATP-binding cassette (ABC) transporters	Walter et al., 2015
*TaPDR1*	Wangshuibai	FJ185035^a^, FJ858380^a^	pleiotropic drug resistance (PDR) type ABC transporter	Shang et al., 2009
*TaPDR7*	Ning 7840	undisclosed	PDR type ABC transporter	Wang et al., 2016
*TaCYP72A*	CM82036	TraesCS3A01G532600^b^	cytochrome P450	Gunupuru et al., 2018
*TaLTP5*	Shannong 0431	JQ652457^a^	lipid transfer protein	Zhu et al., 2012

a GenBank accession number (https://www.ncbi.nlm.nih.gov/nucleotide/).

b Ensembl Plants accession number (http://plants.ensembl.org/Triticum_aestivum/Info/Index).

### Wheat genes whose products are targets of *F. graminearum* effectors

Plant pathogenic microbes always secrete proteins that act as effectors into host cells to evade or inhibit host immunity, leading to enhanced pathogen virulence and facilitated pathogen growth (Dou and Zhou, 2012). The secreted effectors bind host proteins to modify their native biological functions. Some of the host targets are key regulators of plant immunity and therefore could be deployed for disease control.

Secreted proteome of *F. graminearum* has been obtained, with the protein numbers varied in different studies (Yang et al., 2012; Rampitsch et al., 2013; Yang et al., 2013; Lowe et al., 2015). However, their host targets are largely unknown.

It has been found that *F. graminearum* produces orphan secretory proteins (OSPs), and one of them, Osp24, functions as an effector (Jiang et al., 2020). After being secreted into wheat cells, Osp24 binds wheat protein TaSnRK1α (Jiang et al., 2020). The binding by Osp24 accelerates TaSnRK1α degradation, which may suppress host defense responses including cell death and is thus beneficial for pathogen infection; however, physical interaction with TaFROG, a wheat orphan protein, prevents TaSnRK1α from degradation and helps in wheat defense (Jiang et al., 2020). The interplay between the two orphan genes, OSP24 and TaFROG, may be indicative of co-evolution of *F. graminearum* and the host wheat, and the distinctive defense response of wheat to *F. graminearum*.

### Detoxication genes in wheat

The mycotoxins such as deoxynivalenol (DON, a type B trichothecene) produced by the pathogen are toxic to humans and animals. They cause emesis, feed refusal, and even death (Eriksen and Pettersson, 2004). In addition, DON is considered as a virulence factor capable to facilitate disease spread on wheat (Proctor et al., 1995; Bai et al., 2002). *F. graminearum* deficient in DON biosynthesis was able to infect wheat spikelets but failed to spread in spikelets, thus causing diminished disease symptoms (Bai et al., 2002). Therefore, decreasing the amount of DON of wheat grain during pathogen infection is not only necessary for food security, but also one goal of breeding for FHB resistance.

Proteins encoded by various genes have been identified with the ability to detoxify DON (**Table 1**). Among them, uridine diphosphate (UDP)-glycosyltransferases (UGTs) have been widely reported to be able to detoxify DON through glucosylation. These enzymes transfer a glycosyl group from UDP-glucose to DON to conjugate DON into deoxynivalenol-3-O-glucose (D3G), which is nontoxic for animals. As DON can promote disease spreading, glucosylation of DON to D3G is an important plant defense mechanism. He et al. (2018b) systematically analyzed family-1 UGTs and identified 179 putative UGT genes in a reference genome of wheat, Chinese Spring. Among them, *TaUGT3* (Ma et al., 2010b; Pei, 2011; Chen, 2013), *TaUGT5* (Zhao et al., 2018), and *TaUGT6* (He et al., 2020) were validated to be effective in reducing DON content in wheat. Wheat lines overexpressing the three genes respectively showed resistance to DON treatment and the resultant disease resistance to FHB, implying the potential of *TaUGT* as useful disease resistance genes in breeding for FHB resistance.

Adenosine triphosphate (ATP)-binding cassette (ABC) transporters have been implicated in DON detoxication. They may export DON from the cytoplasm to reduce the damage caused by mycotoxin. *TaABCC3*, encoding an ABC transporter responsible for substance transport across cell membrane, was cloned from DON-treated wheat transcripts (Walter et al., 2015). Inhibition of *TaABCC3* expression by VIGS increased wheat sensitivity to DON (Walter et al., 2015). However, the effect of *TaABCC3* on FHB resistance was not analyzed. *TaPDR1* and *TaPDR7*, two wheat genes encoding the pleiotropic drug resistance (PDR) subfamily of ABC transporters, were upregulated by DON treatment and *F. graminearum* infection; knockdown of *TaPDR7* in wheat by VIGS compromised FHB resistance (Shang et al., 2009; Wang et al., 2016).

Cytochrome P450, membrane-bound enzymes that can perform several types of oxidation–reduction reactions, was also reported to possess the ability to catabolize DON (Ito et al., 2013). A wheat P450 gene, *TaCYP72A*, was found to be activated by DON treatment and *F. graminearum* infection (Gunupuru et al., 2018). Suppressing *TaCYP72A* through VIGS reduced wheat resistance to DON (Gunupuru et al., 2018). However, whether this gene confers FHB resistance is not identified.

## Exploration and utilization of alien genes in *Triticeae* with FHB resistance

There are over 300 species classified under more than 20 genera in *Triticeae* (Dewey, 1984), which represent an invaluable gene pool for wheat improvement. The wild relatives of wheat are an important source for wheat improvement with FHB resistance. Many genes with FHB resistance have been identified and verified *in vivo* (**Table 2**). They show unique features as well as shared characteristics with those identified in hexaploidy wheat.

**Table 2:** Genes identified with FHB resistance in wheat relatives.

Species	Gene name	Accession number	Gene products	References
*Thinopyrum elongatum*	*Fhb7*	Tel7E01T1020600.1^a^	glutathione S-transferase	Wang et al., 2020a
*Hordeum vulgare*	*tlp-1*	AM403331^b^	thaumatin-like protein	Mackintosh et al., 2007
	*PR2*	M62907.1^b^	β‐1,3‐glucanase	Mackintosh et al., 2007
	*HvWIN1*	KT946819^b^	ethylene-responsive transcription factor	Kumar et al., 2016
	*HvLRRK-6H*	MLOC_12033.1^c^	receptor like kinase	Thapa et al., 2018
	*chitinase gene*	M62904^b^	chitinase	Shin et al., 2008
	*HvUGT13248*	GU170355^b^	uridine diphosphate-glycosyltransferase	Schweiger et al., 2010; Shin et al., 2012; Li et al., 2015; Li et al., 2017; Mandalà et al., 2019
	*HvUGT-10W1*	undisclosed	uridine diphosphate-glycosyltransferase	Xing et al., 2017
*Brachypodium distachyon*	*Bradi5g03300*	Bradi5g03300	uridine diphosphate-glycosyltransferase	Schweiger et al., 2013; Pasquet et al., 2016; Gatti et al., 2019
	*BdCYP711A29*	Bradi1g75310	cytochrome P450 monooxygenase	Changenet et al., 2021
*Haynaldia villosa*	*CERK1-V*	Dv07G125800	chitin-recognition receptor	Fan et al., 2022

a WheatOmics accession (http://wheatomics.sdau.edu.cn).

b GenBank accession number (https://www.ncbi.nlm.nih.gov/nucleotide/).

c IPK Barley Blast Server (http://webblast.ipk-gatersleben.de/barley).

### Genes from *Thinopyrum*

Of the QTL that showed a stable major effect on FHB resistance, *Fhb7* was transferred from wheatgrass *Thinopyrum* (Fu et al., 2012; Guo et al., 2015), and was cloned recently using the map-based cloning approach (Wang et al., 2020a). *Fhb7* was mapped to chromosome 7E of *Th. elongatum* (Fu et al., 2012; Guo et al., 2015). The underpinning gene of the locus was identified that encodes a glutathione S-transferase (GST) with the prominent ability to detoxify trichothecene toxins produced by the pathogens (Wang et al., 2020a). The expression of the gene was increased at the late stage of infection and was also induced by trichothecene treatment (Wang et al., 2020a), implying an active role of *Fhb7* in the response to mycotoxins. How *Fhb7* is regulated at the molecular level remains obscure and needs to be determined. This may help to increase the expression of *Fhb7* in wheat cultivars for further enhanced FHB resistance. Notably, wheat lines with *Fhb7* locus showed increased resistance to FHB without growth defect and yield penalty (Wang et al., 2020a), making *Fhb7* a promising potential for wheat resistance breeding.

### Genes from *Hordeum vulgare*

Barley (*Hordeum vulgare*) *PR* genes also contributed to FHB resistance. Transgenic wheat lines separately overexpressing barley *tlp-1* and *PR2* gene showed increased resistance to FHB (Mackintosh et al., 2007). Two other genes, *HvWIN1* encoding a transcriptional regulator of cuticle biosynthetic genes and *HvLRRK-6H* encoding a leucine-rich receptor-like kinase, were identified as positive regulators of FHB resistance (Kumar et al., 2016; Thapa et al., 2018). Knockdown of the two genes individually by VIGS increased the disease severity of barley (Kumar et al., 2016; Thapa et al., 2018). Wheat lines overexpressing a barley chitinase gene improved wheat resistance to FHB (Shin et al., 2008).

UGT genes responsible for DON detoxication have also been identified in barley. *HvUGT13248* played an effective role in DON detoxication when expressed in yeast (Schweiger et al., 2010), *Arabidopsis* (Shin et al., 2012), durum (Mandalà et al., 2019), and wheat (Li et al., 2015; Li et al., 2017; Mandalà et al., 2019). Wheat lines constitutively expressing *HvUGT13248* showed improved FHB resistance (Li et al., 2015; Li et al., 2017; Mandalà et al., 2019).

Recombinant HvUGT-10W1 purified from bacterium cells inhibited hypha growth of *F. graminearum* in the PDA (potato/dextrose/agar) media. Furthermore, suppressing *HvUGT-10W1* expression in a barley variety 10W1, which showed resistance to FHB, using the VIGS approach reduced the resistance to FHB (Xing et al., 2017), implying the positive role of *HvUGT-10W1* in barley resistance to FHB.

### Genes from *Haynaldia villosa*

*CERK1-V* (Dv07G125800), the chitin-recognition receptor of *Haynaldia villosa*, was recently cloned and introduced into wheat under the drive of maize ubiquitin promoter (Fan et al., 2022). The overexpression lines showed enhanced FHB resistance, implying that the perception of chitin is an important step to initiate FHB resistance. Therefore, it has potential to identify the genes involved in chitin signaling and develop them for FHB resistance.

### Genes from *Brachypodium distachyon*

*Brachypodium distachyon* has been developed for FHB resistance analysis (Peraldi et al., 2011). Several genes from *B. distachyon* have been characterized by FHB resistance. *Bradi5g03300 UGT* gene has been introduced into *B. distachyon* Bd21-3 and the wheat variety Apogee, both of which are susceptible to FHB (Schweiger et al., 2013; Pasquet et al., 2016; Gatti et al., 2019). Enhanced resistance to FHB and strong reduction of DON content in infected spikes were observed in the transgenic lines. Promisingly, some of the transgenic lines with high *Bradi5g03300* transcripts showed normal growth or phenotype compared with the wild type.

*BdCYP711A29* (Bradi1g75310) encoding cytochrome P450 monooxygenase involved in orobanchol (one form of strigolactones) biosynthesis was identified to negatively regulate FHB resistance. Overexpression of *BdCYP711A29* in *B. distachyon* increases susceptibility to FHB, while the TILLING mutants showed disease symptoms similar to those of the wild type (Changenet et al., 2021).

## FHB resistance genes from plant species beyond *Triticeae* species

### Genes from *Arabidopsis thaliana*

*Arabidopsis* has been exploited for the analysis of the scientific rationale of plant resistance to *F. graminearum* because the fungi can infect *Arabidopsis* flowers (Urban et al., 2002; Brewer and Hammond-Kosack, 2015). Many genes that have been identified from *A. thaliana* showed potential for resistance against these pathogenic fungi.

NPR1 is an ankyrin repeat-containing protein involved in the regulation of systemic acquired resistance. Wheat lines overexpressing NPR1 showed enhanced resistance to FHB (Makandar et al., 2006).

AtALA1 and AtALA7, two members of *Arabidopsis* P-type ATPases, contributes to plant resistance to DON through cellular detoxification of mycotoxins (Wang et al., 2021). They mediated the vesicle transport of toxins from the plasma membrane to vacuoles. Transgenic *Arabidopsis* or maize plants overexpressing *AtALA1* enhanced resistance to DON and disease caused by *F. graminearum*. It remains unknown whether *AtALA1* homologous genes exist in wheat genome and have the same detoxification function.

### Genes from rice and maize

*F. graminearum* also infects other crops, such as rice (*Oryza sativa* L.) and maize (*Zea mays* L.). In rice plants, a UGT OsUGT79 expressed and purified from bacterium cells was reported to be effective in conjugating DON into D3G *in vitro* (Michlmayr et al., 2015; Wetterhorn et al., 2017) and could be used as a promising candidate for FHB resistance breeding. Additionally, overexpression of rice *PR5* gene encoding thaumatin‐like protein in wheat reduced FHB symptoms (Chen et al., 1999).

In maize, *RIP* gene *b-32* encoding ribosome inactive protein promotes FHB resistance when overexpressed in wheat (Balconi et al., 2007).

## Host-induced and spray-induced gene silencing of genes in *Fusarium graminearum*

Host-induced gene silencing (HIGS) was recently developed to control fungal diseases, in which transgenic host plants produce small interference RNAs (siRNAs) that match important genes of the invading pathogen to silence fungal genes during infection (Machado et al., 2018). Koch et al. (2013) reported that detached leaves of both transgenic *Arabidopsis* and barley plants expressing double-stranded RNA from cytochrome P450 lanosterol C-14a-demethylase genes exhibited resistance to *F. graminearum*. HIGS transgenic wheat targeting the chitin synthase 3b also confers resistance to FHB (Cheng et al., 2015). HIGS targeting multiple genes involving *FgSGE1*, *FgSTE12*, and *FgPP1* of the fungus is effective and can be used as an alternative approach for developing FHB- and mycotoxin-resistant crops (Wang et al., 2020b).

Spray-induced gene silencing (SIGS), mechanistically similar to HIGS, is also effective to fungal disease control. In this approach, sprayed siRNAs or noncoding double-stranded (ds)RNAs onto plant surfaces targeting key genes of pathogens are taken up by the pathogens and in turn inhibit pathogen gene expression, leading to inhibited pathogen growth in plants (Koch et al., 2016; Song et al., 2018). SIGS targeting *F. graminearum* genes has been reported to effectively inhibit the pathogen growth in barley, providing the potential for FHB control (Koch et al., 2019; Werner et al., 2020; Rank and Koch, 2021).

## Challenges and perspectives

**Figure 1:**
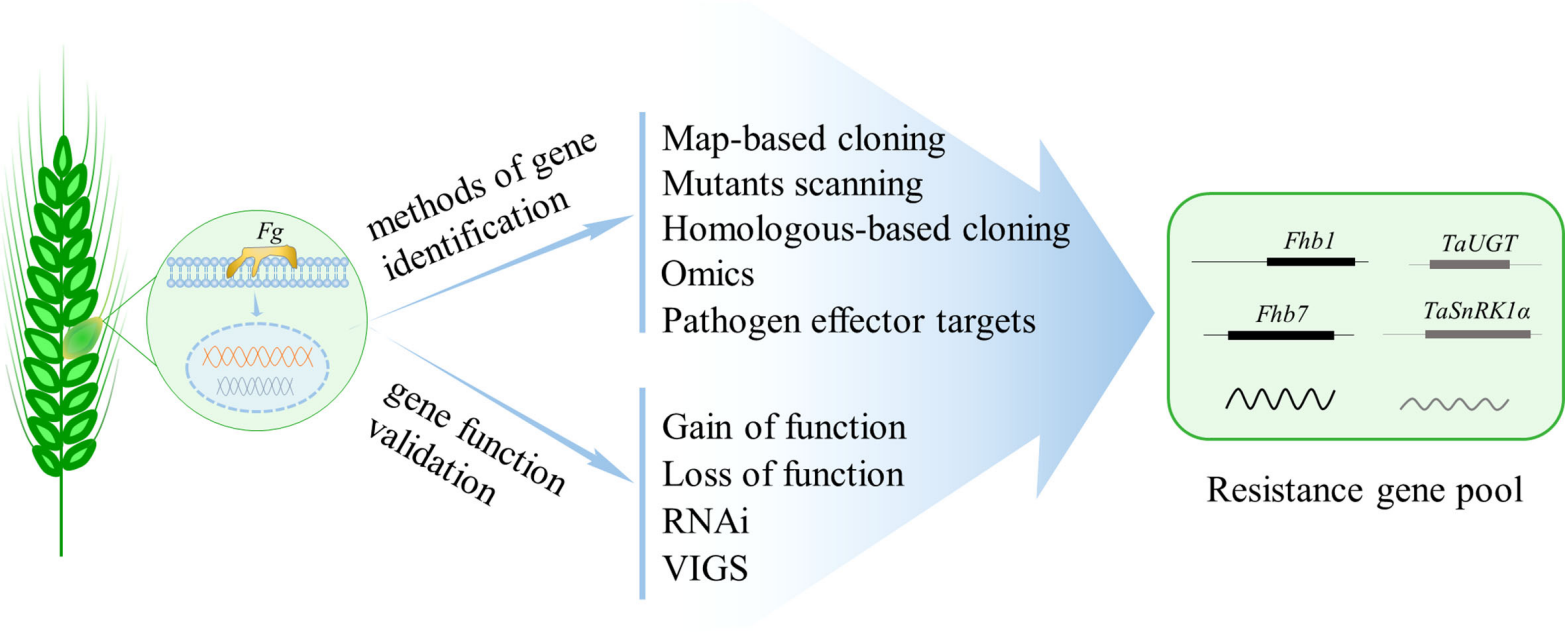
Methods to identify and validate genes that show resistance to *Fusarium graminearum* (*Fg*) and compose the resistance gene pool for wheat FHB resistance breeding. Map-based cloning and mutants scanning are two widely used approaches in gene identifying, while the latter is rarely applied in the analysis of wheat FHB resistance partially due to the functional redundancy among homeologs in polyploid wheat. With the completion of wheat genome sequencing, the popularized reverse-genetic approaches such as homologous-based cloning and omics technology are capable of identifying FHB resistance genes. Additionally, identifying the targets of pathogen effectors is also effective in wheat resistance gene identification. Finally, various gene gain- or loss-of-function technologies, or gene knockdown approaches such as RNAi and VIGS, are applicable to validate gene function in wheat FHB resistance.

Compared with agronomic practices, chemical control, and biological control, genetic resistance is the best and most cost-effective strategy that could provide meaningful, consistent, and durable FHB control (Shude et al., 2020). There are variations in the susceptibility of different host plant species to FHB; however, no wheat varieties possess immunity against FHB (Dweba et al., 2017). Though hundreds of QTL have been reported in wheat, only two, *Fhb1* and *Fhb7*, have been cloned through years of hard work (Rawat et al., 2016; Li et al., 2019; Su et al., 2019; Wang et al., 2020a); thus, the FHB resistance genes that can be used for breeding is obviously limited. How to improve FHB resistance to a high level in wheat using the limited genes is a fundamental ongoing challenge. Pyramiding resistance genes to increase FHB resistance is feasible and popularized. However, the strategy is highly dependent on the adequate resistance genes. The majority of the QTL identified usually show a minor effect on FHB resistance and have no diagnostic markers. Therefore, sustained and continuous efforts are still needed in cloning and validating resistance genes from hundreds of QTL associated with FHB resistance in wheat. The integration of various forward- and reverse-genetic approaches will be an important means to explore the genes of FHB resistance in wheat, with the development of plant–pathogen interaction mechanism in model plants (**Figure 1**).

In contrast to FHB resistance genes, modification of the susceptibility (S) genes will be an alternative option for controlling FHB. S factors or resistance suppressors have already been located on different chromosomes in wheat, yet, to date, they have not received much attention (Ma et al., 2006). In plants, some host factors encoded by S genes are always hijacked by pathogens through the secreted effectors to promote disease development. Mutations of these S genes evade the manipulation by pathogens and have been successfully utilized in crop disease control including wheat resistance to fungal pathogen (Garcia-Ruiz et al., 2021; Koseoglou et al., 2022). As secreted proteome of *F. graminearum* has been obtained, in depth analysis of the interaction between host and *F. graminearum* would facilitate the understanding of the function of S genes in wheat. Genes encoding resistance suppressors that always inhibit plant immune responses fall into another category of S genes (Garcia-Ruiz et al., 2021; Koseoglou et al., 2022). Mutations of these genes with abolished or reduced gene expression generally result in enhanced and durable disease resistance. These genes are significant components of crop disease resistance gene pool, while in FHB resistance, no such genes have been isolated so far. With the development of wheat mutant libraries, identification of such S genes will be facilitated.

Though some genes with FHB resistance have been identified, the signaling pathway that wheat perceives and responds to *F. graminearum* attack remains obscure. Plant immunity involves large-scale changes in gene expression. Intensive investigation of the role of those genes responsive to *F. graminearum* in wheat FHB resistance will contribute to the cloning of practical resistance genes. With the completion of wheat genome sequencing, the initiation of pan-genomic research for tribe *Triticeae*, and the rapid development in biotechniques, breakthrough will be made in the field in the future.

## Author contributions

HGM and HXM wrote the manuscript. YL, XZ and SZ collected some data. All authors contributed to the article and approved the submitted version.

## Funding

This research was funded by the Jiangsu Key Project for the Research and Development (BE2022337) and the Seed Industry Revitalization Project of Jiangsu Province (JBGS2021047).

## Conflict of interest

The authors declare that the research was conducted in the absence of any commercial or financial relationships that could be construed as a potential conflict of interest.

## Publisher’s note

All claims expressed in this article are solely those of the authors and do not necessarily represent those of their affiliated organizations, or those of the publisher, the editors and the reviewers. Any product that may be evaluated in this article, or claim that may be made by its manufacturer, is not guaranteed or endorsed by the publisher.
